# 2074: Comparing the properties of human umbilical cord-derived mesenchymal stromal cells from preterm Versus full-term infants

**DOI:** 10.1017/cts.2017.196

**Published:** 2018-05-10

**Authors:** Alvaro Moreira, Caitlyn Winter, Saloni Balgi, Shamimunisa Mustafa, Lauryn Winter, Peter Hornsby

## Abstract

OBJECTIVES/SPECIFIC AIMS: To compare functional differences in WJ-MSCs-derived from term Versus preterm infants. METHODS/STUDY POPULATION: WJ-MSCs were enzymatically digested from umbilical cord tissue from Term (gestational age ≥37 wk, n=4) and Preterm (gestational age ≤32 wk, n=5) neonates. Cells were characterized by (1) surface antigen markers using flow cytometry, (2) ability to differentiate into adipogenic, chondrogenic, and osteogenic lineages following in vitro stimulation, (3) colony forming unit efficiency, (4) proliferation rates, and (5) cell motility assay. RESULTS/ANTICIPATED RESULTS: WJ-MSCs were successfully isolated from both Preterm and Term groups. Cells adhered to plastic and displayed characteristic spindle-shaped morphology when cultured under standard conditions. WJ-MSCs from both groups expressed surface antigen markers CD73, CD90, and CD146 (≥90%) and did not express hematopoietic markers HLA-DR, CD79, or CD117 (<5%). Preterm and Term cells were capable of differentiating into osteogenic, chondrogenic, and adipogenic lineages. There were no significant differences between the groups when evaluated by colony forming efficiency, proliferation rates, or cell motility. DISCUSSION/SIGNIFICANCE OF IMPACT: These preliminary findings suggest that WJ-MSCs derived from full-term or preterm neonates have similar functional characteristics. Future studies will focus on the regenerative potential of WJ-MSCs from preterm and term infants following changes in the microenvironment (eg, oxygen tension, inflammatory stimulation).

